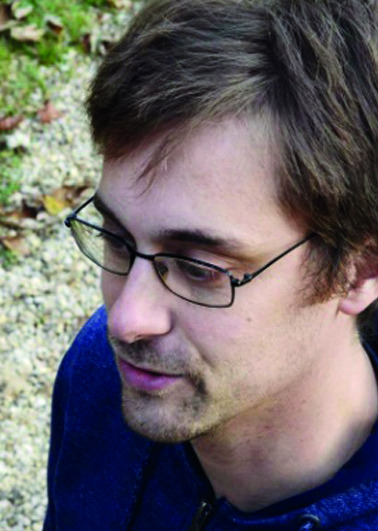# Tyrosinase Inhibition and Antimelanogenic Effects of Resorcinol‐Containing Compounds

**DOI:** 10.1002/cmdc.202400314

**Published:** 2024-09-30

**Authors:** Morane Beaumet, Leticia M. Lazinski, Marc Maresca, Romain Haudecoeur

**Affiliations:** ^1^ Univ. Grenoble Alpes CNRS DPM 38000 Grenoble France; ^2^ Univ. Grenoble Alpes CNRS DCM 38000 Grenoble France; ^3^ Aix Marseille Univ. CNRS Centrale Marseille iSm2 13013 Marseille France

**Keywords:** Tyrosinase, Metalloenzymes, Melasma, Melanogenesis, Medicinal Chemistry

## Abstract

Tyrosinases (TYRs) are copper‐containing metalloenzymes present in a large diversity of species. In human, hTYR is responsible for pivotal steps in melanogenesis, catalysing the oxidation of l‐tyrosine to l‐DOPA and further to dopaquinone. While numerous TYR inhibitors have been reported, polyphenolic compounds tend to dominate the literature. However, many of these compounds, particularly monophenols and catechols, have been identified as alternative substrates rather than true inhibitors, given their structural similarity to natural substrates. Resorcinol‐containing compounds have emerged as promising candidates to address this challenge, as the *meta*‐dihydroxy moiety in resorcinol demonstrates resistance to TYR‐mediated oxidation, while retaining the favourable interactions with copper ions provided by the hydroxy groups. Although their precise mechanism of action remains debated, resorcinol derivatives have yielded some of the most active compounds against isolated mushroom and human TYRs, as well as clinically used dermocosmetic agents like rucinol and thiamidol, which exhibited very promising effects in patients with facial melasma. This review outlines the development of resorcinol‐containing TYR inhibitors, categorized by scaffold type, ranging from simple alkyl analogues to intricate synthetic derivatives. Mechanistic insights about the resorcinol‐TYR interaction are also presented and debated.

## Graphical abstract

Resorcinol‐based compounds stand out as key inhibitors of tyrosinases (TYR) owing to their phenolic group's interaction with the dicopper centre and their ability to resist TYR‐mediated oxidation. While some well‐known examples are employed in dermatocosmetics and skin‐whitening products, this review covers the broader diversity of scaffolds, including inhibition mechanisms, preclinical and clinical data, and molecular modes of action.

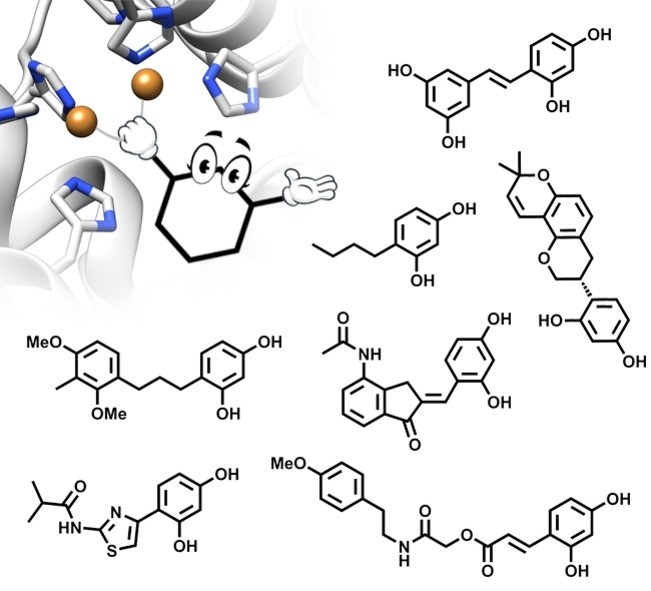

## 1 Introduction

**Figure 1:**
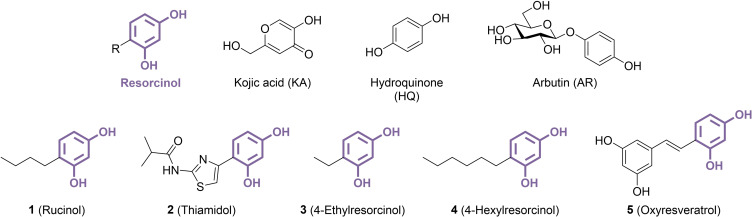
Structure of the resorcinol moiety, TYR inhibitors used as reference in most studies, and most known resorcinol‐based TYR inhibitors.

Tyrosinases (TYRs, EC 1.14.18.1) are type‐3 copper‐containing metalloenzymes, characterized by a coupled binuclear centre coordinated by six highly conserved histidine residues. They catalyse both the *ortho*‐hydroxylation of monophenols and the oxidation of catechols into *ortho*‐quinones, providing a central catalytic activity in various melanogenesis pathways and enabling key physiological functions for many organisms, including bacteria and fungi, where TYR activity appears as essential for pathogenicity and virulence,[^1^, ^2^] as well as vertebrates.[^3^, ^4^] In mammals, and especially in humans, TYR carries out the rate‐limiting l‐tyrosine double oxidation step in the biosynthesis of eumelanin and pheomelanin, which are crucial pigments for skin protection against UV radiation and environmental aggressions.^[5]^ Hence, TYR has garnered significant attention as the pivotal enzyme controlling human melanogenesis, making it a prime target for treatments aimed at managing hyperpigmentation^[6]^ or facilitating skin‐whitening applications. However, historically, many TYR inhibitors were developed using copper‐interacting moieties inspired directly from natural substrates l‐tyrosine and l‐DOPA. Consequently, monophenol‐ and catechol‐based TYR‐targeting compounds abound in the literature, yet a significant proportion of them function as alternative substrates rather than inhibitors.^[7]^ Indeed, TYR exhibits remarkable tolerance to a wide range of phenolic substrates, as long as they present a compatible oxidation state. One alternative strategy has been the development of resorcinol analogues (mostly defined herein as 2,4‐dihydroxyphenyl derivatives, Figure 1), which usually demonstrate greater resistance to TYR‐mediated oxidation due to the presence of a *meta*‐dihydroxy pattern. In addition, while mushroom TYR (mTYR) has been widely used to discover inhibitors, its low homology with human TYR (hTYR) caused misleading findings, and genuine hTYR inhibitors emerged only recently.^[8]^ The development of resorcinol‐containing compounds like rucinol (**1**) and thiamidol (**2**) successfully overcame both challenges at once, exhibiting significantly improved efficacy and safety profiles compared to traditional agents like kojic acid (KA), hydroquinone (HQ) and arbutin (AR, Figure 1), and making them valuable agents widely used in dermocosmetics. Other derivatives were found to inhibit mTYR, suggesting potential applications in the agrofood industry where TYR activity leads to undesirable tissue browning in fruits and vegetables.^[9]^ In summary, this review delves into the potential of resorcinol‐containing compounds as antimelanogenic agents, exploring their effectiveness both *in vitro* and *in vivo*, along with their molecular diversity and mechanism of action, which continues to be a topic of debate.

## 2 Controversial Action of the Resorcinol Moiety on TYR

**Figure 2:**
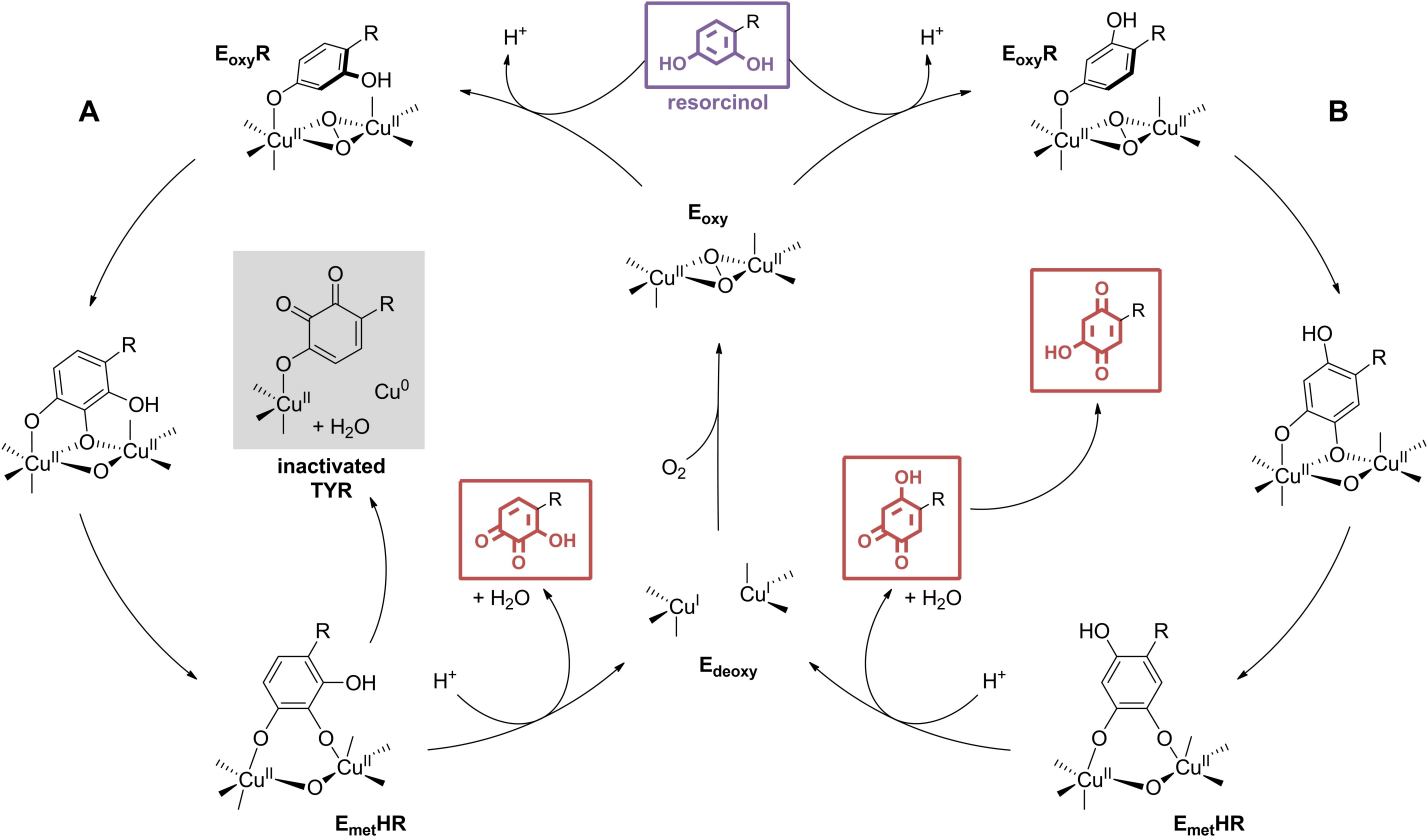
Two reported mechanistic hypotheses for resorcinol action on TYR, alternative to reversible and non‐covalent inhibition: suicide inactivation (A) and secondary substrate oxidation (B).

Initially presumed to be TYR inhibitors, resorcinol derivatives have undergone extensive scrutiny over nearly two decades, with successive studies characterizing them as suicide inactivators, secondary substrates or genuine inhibitors. In a first report, unlike primary catechol‐based substrates that require oxygen consumption for TYR‐mediated oxidation, 4‐ethylresorcinol (**3**) and analogues demonstrated inactivation properties independent of oxygen utilization.^[10]^ This suggests that resorcinols may adopt a monophenolase‐like presentation at the dicopper centre, inactivating TYR through the elimination of a Cu^0^ atom, akin to the Quintox mechanism reported during unconventional monooxygenase action on catechols (Figure 2A).^[11]^ Further experiments provided evidence supporting a suicide inactivation by resorcinols, as the inhibition of TYR diphenolase activity was observed to vary with the incubation time, and LC–MS profiling identified products of resorcinol monophenolase oxidation, particularly 3‐hydroxy‐*ortho*‐quinones, that were not detected in the previous study.^[12]^ In addition, the contributions of substituents were found to be non‐additive, contrary to what would be expected for reversible inhibitors. However, the authors acknowledged that the findings with the simple resorcinol derivatives studied does not necessarily imply a similar mechanism of action for more complex analogues capable of establishing multiple non‐covalent interactions at the second sphere of coordination in the active site pocket. Subsequently, another research group investigated the effects of resorcinols on TYR as alternative substrates. Several successive reports described the oxidation of 4‐hexylresorcinol (**4**),^[13]^ oxyresveratrol (**5**),^[14]^ rucinol (**1**),^[15]^ resorcinol, 4‐ethylresorcinol (**3**) and 4‐methylresorcinol^[16]^ by TYR (Figure 1). However, in each case, these compounds remained unaffected when exposed to the native state of the enzyme, which primarily exists as the E_met_ form,^[17]^ with typically 7–15 % in the E_oxy_ form.^[18]^ The artificial prior generation of the E_oxy_ form through treatment with H_2_O_2_ or ascorbic acid was required to observe changes in the absorption spectra. The appearance of a band around 480 nm was especially indicative of *para*‐quinone formation, likely by *ortho*‐quinone isomerization following catalytic oxidation (Figure 2B). In addition, it was stated that resorcinols do not inactivate TYR, as TYR samples incubated with **3** recovered their initial activity levels after filtration through a Sephadex column. Notably, all the resorcinols studied exhibited kinetic parameters consistent with a very slow oxidation process, with k_cat_ values ranging from 0.8 to 20 s^−1^, which is 10− to 100‐fold lower than monophenol counterparts. This TYR‐mediated oxidation phenomenon did not appear to occur with all resorcinols however. For instance, 2,2’,4,4’‐tetrahydroxybenzophenone, examined by the same group, exhibited weak TYR inhibition properties and did not react with the enzyme in similar conditions.^[19]^ Another study contradicted the potential substrate role of rucinol by investigating mTYR‐mediated oxidation of various phenolic compounds.^[20]^ While most monophenols underwent oxidation, as confirmed by UV‐visible and HPLC analyses, rucinol was mostly unaffected by TYR. Yet, both monophenols and resorcinols would require the formation of the E_oxy_ form before reacting with TYR, as proposed in the alternative substrate hypothesis. Furthermore, the presence of l‐DOPA, which is known to increase the proportion of E_oxy_ form,^[21]^ significantly accelerated the initially slow oxidation of 4‐*tert*‐butylphenol by TYR, whereas it did not produce any visible effect on rucinol. The authors more recently provided additional insights through metabolite analysis in human tyrosinase‐expressing non‐melanogenic 293 T cells treated with phenolic compounds.^[22]^ All the tested monophenols and catechols previously reported as leukoderma‐inducing agents were oxidized by TYR, as evidenced by the detection of physiological thiol adducts of the corresponding *ortho*‐quinones and a simultaneous decrease in cellular GSH levels. In contrast, rucinol did not produce similar metabolites or GSH depletion, indicating its resistance to TYR catalytic activity in a cell‐based context. In another study, monophenolic derivatives 4‐hydroxyphenol and raspberry ketone showed TYR‐induced cytotoxicity in B16 melanogenic melanoma cells, leading to vacuolization, while **1** did not exert any significant impact on cell viability.^[23]^ This suggests that while leukoderma induced by monophenols may stem from toxic *ortho*‐quinone production due to TYR catalytic activity, the lack of effect observed with **1** implies that this compound, along with potentially other resorcinol derivatives, acts as a genuine inhibitor in cellular assays. In conclusion, the mechanism of action of resorcinols remains contentious and appears to vary among different molecules and assays. Comprehensive studies, extending beyond a limited set of simple resorcinols, are still necessary to elucidate the observed effects and understand their precise interactions with TYR and the consequences for applications in dermocosmetic and food‐related contexts. However, resorcinols targeting TYR discussed in this review will be referred to as inhibitors.

## 3 Resorcinol‐Containing Compounds as TYR Inhibitors

In the literature, nearly all reported TYR inhibitors are evaluated using spectrophotometric methods. These enzymes can oxidize a wide range of substrates, including the native ones l‐tyrosine and l‐DOPA, both commonly used in inhibition assays. However, since TYR's oxidation of monophenols and diphenols follows distinct pathways, involving different states of the dicopper centre and different kinetic parameters (such as the presence of a ‘lag’ phase in monophenolase activity), the results obtained are often difficult to compare directly. Therefore, for clarity, each inhibition value presented below is specified along with the substrate used in the test and the corresponding value recorded for a known reference inhibitor, most often kojic acid (KA).

### 3.1 Rucinol, Phenylethyl Resorcinol and Other Simple 4‐Alkylresorcinols

**Figure 3:**
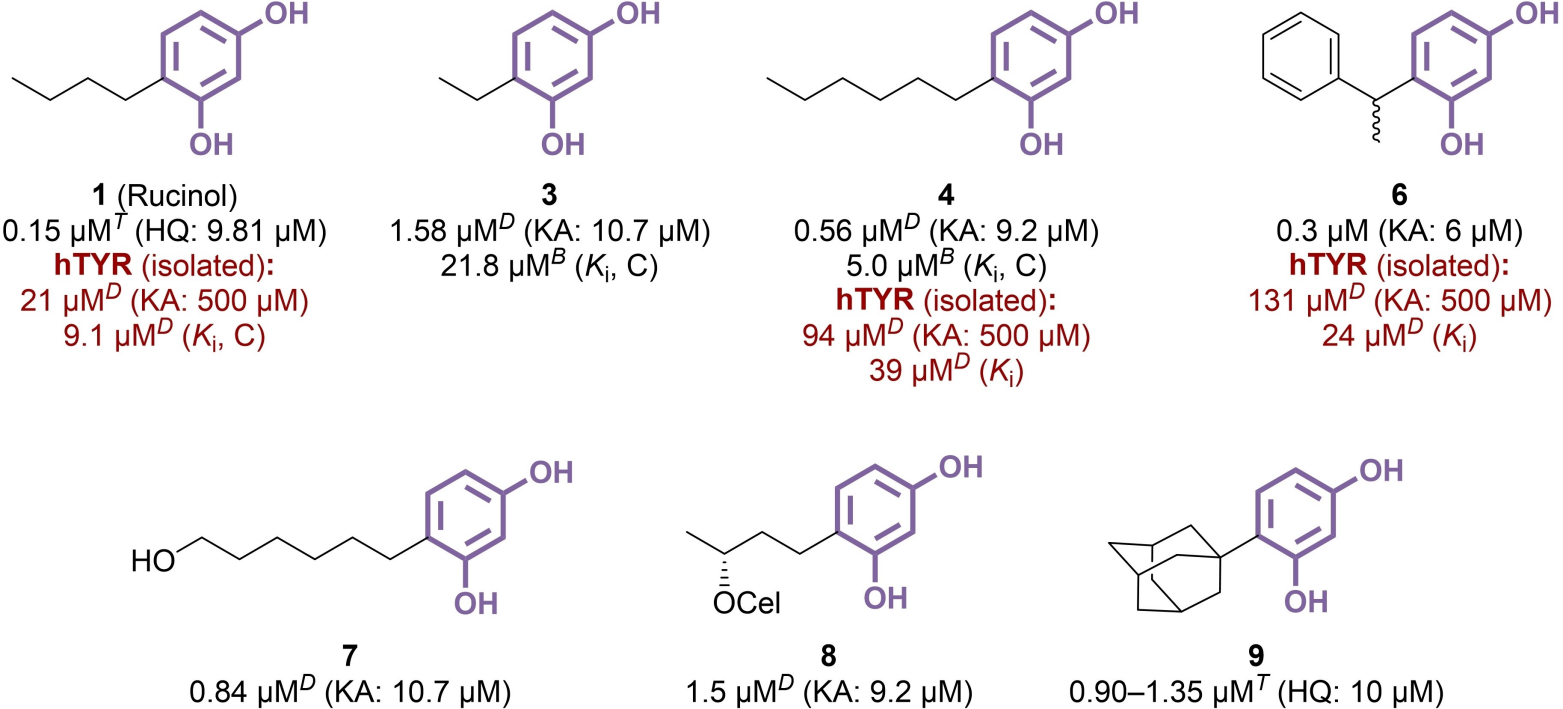
4‐Alkylresorcinol derivatives as TYR inhibitors (C: competitive inhibition, *T*: l‐tyrosine used as substrate, *D*: l‐DOPA used as substrate, *B*: 4‐*t*‐butylphenol used as substrate).

Commercialized in Japan as a ‘quasi‐drug’ in 1998,^[24]^ rucinol (**1**) is an emblematic example of TYR‐targeting skin whitening agent, still widely used in dermocosmetics, along with two other 4‐alkylresorcinols, *i. e*., 4‐hexylresorcinol **4** and 4‐phenylethylresorcinol **6** (Figure 3). These compounds are widely considered safe for cosmetic use, particularly in comparison to harmful substances like kojic acid and hydroquinone, which have been prohibited by regulations in many regions. All three 4‐alkylresorcinols exhibited competitive inhibition against mTYR with potencies below the micromolar range (IC_50_=0.15–0.56 μM),[^25^, ^26^, ^27^, ^28^, ^29^] while results obtained from hTYR presented a more nuanced picture (IC_50_ =21–131 μM).[^28^, ^30^] Rucinol especially yielded an IC_50_ value of 8.3 μM against hTYR activity in human melanoma MNT‐1 cell lysates,^[31]^ and further examination of its cellular mechanism in murine Mel‐Ab melanocytes revealed a direct inhibition of TYR catalytic activity, excluding regulation *via* ERK, Akt, or CREB pathways.^[32]^ In addition, if no effects were recorded on TYR glycosylation and trafficking in murine B16F10 cells, rucinol enhanced the proteolytic degradation of TYR through p38 MAPK activation.^[33]^ At the clinical level, several studies investigated the effect of rucinol‐containing creams against melasma in randomized split‐face trials. First, the application of a 0.3 % rucinol serum in 32 patients with facial melasma provided significant reduction of the clinical pigmentation score after 12 weeks, compared to treatment with the vehicle alone.^[34]^ Subsequent works aimed to reduce rucinol concentration to 0.1 %, with promising outcomes: after 8 weeks, the melanin index of patients with melasma decreased significantly, both without (−4.87 %) and with (−7.51 %) liposome encapsulation.[^35^, ^36^] Throughout these studies, rucinol was well‐received by participants, causing only mild and transient side effects, including occasional itching, stinging, burning or peeling. Similarly, 4‐hexylresorcinol demonstrated efficient melanogenesis inhibition in human melanocytes with negligible toxicity,^[37]^ and in clinical trials, the application of a 1 % 4‐hexylresorcinol preparation on patients resulted in a notable reduction in pigmentation after 4 and 12 weeks, comparable to the effects observed with 2 % hydroquinone treatments.^[38]^ Regarding 4‐phenylethylresorcinol, although a 0.5 % dosage resulted in successful skin lightening,^[39]^ its limited solubility and photostability, coupled with its tendency to induce allergic contact dermatitis,^[40]^ hindered its suitability for topical applications, despite numerous attempts at formulation improvement using nanoparticles.[^41^, ^42^, ^43^] However, beyond these cosmetic agents, other alkyl derivatives have been explored for their potential to inhibit TYR activity. Overall, variations in the length of linear alkyl side chains had minimal impact on the recorded activities (IC_50_=0.53–0.85 μM for C3 to C14 analogues), except for **3**, which demonstrated slightly lower efficiency (IC_50_=1.58 μM), as confirmed by kinetic constant determination using unnatural substrate 4‐*t*‐butylphenol (*K*_i_=21.8 μM for **3**, *vs*. 5.0 μM for **4**).^[44]^ The addition of hydroxy or *O*‐glucosyl groups at the terminal end did not significantly alter the IC_50_ values for longer chains (*e. g*., 0.84 μM for **7** *vs*. 0.56 μM for **4**) but adversely affected shorter chains, particularly C2 (IC_50_=9.3 μM and 36 μM *vs*. 1.58 μM) and C3 (IC_50_=2.2 μM and 3.6 μM *vs*. 0.79 μM).[^29^, ^45^] In contrast, introducing various glycosides at position 3 of the butyl chain of rucinol was globally detrimental, regardless of the resulting chiral centre being *R* or *S* configuration. The most favourable outcome was observed with *R* cellobioside **8**, which had an IC_50_ of 1.5 μM.[^46^, ^47^] Lastly, while TYR tolerated long linear alkyl groups, it was found that a bulky adamantyl moiety also fit well within the binding pocket, as evidenced by the similar values measured for compound **9** (IC_50_=0.90–1.35 μM).[^25^, ^48^] These findings provided valuable insights into the permissible extent of side chain modifications without encountering major steric clash issues, laying the groundwork for the development of larger and more intricate derivatives.

### 3.2 Cinnamic Acids and Derivatives

**Figure 4:**
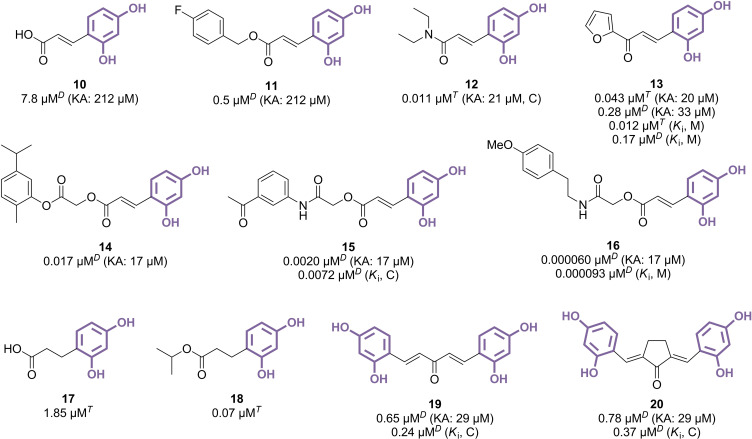
Cinnamic acid derivatives as TYR inhibitors (C: competitive inhibition, M: mixed‐type inhibition, *T*: l‐tyrosine used as substrate, *D*: l‐DOPA used as substrate).

Building on the foundation of previously detailed 4‐alkylresorcinols, a range of cinnamic acid derivatives featuring a 2,4‐dihydroxyphenyl motif were developed to target TYR inhibition (Figure 4). However, the intrinsic structure of cinnamic acid itself exhibited only modest inhibitory effects against mTYR (IC_50_=7.8 μM for compound **10**), likely due to the enzyme's active site favouring hydrophobic interactions over polar protic groups. Efforts to esterify these molecules proved to be much more effective, significantly enhancing their inhibitory potential by 10 to 15 times and achieving submicromolar levels of activity, as demonstrated by the fluoro‐substituted compound **11** (IC_50_=0.5 μM).^[49]^ The formation of amido counterparts was also explored with diverse *N*‐substitutions, encompassing cyclic pyrrolidine‐, piperidine‐, morpholine‐ and piperazine‐based amides, along with simpler alkyl or phenyl variations.[^50^, ^51^, ^52^] Of these derivatives, diethyl compound **12** emerged as particularly effective, showing potent inhibition against isolated mTYR (IC_50_=0.011 μM) and reducing melanogenesis in murine B16F10 melanoma cells. Connecting thiophene and furan rings directly to the cinnamic carbonyl yielded similar outcomes (IC_50_=0.043 μM for **13**),[^53^, ^54^] confirming the possibility of establishing extensive interactions with TYR at this side of the cinnamic acid scaffold, and setting the stage for further investigations into related chalcones. Compound **13** was found as a mixed‐type inhibitor however, suggesting several binding sites. Longer chains were proposed to capitalize on the steric tolerance of mTYR. Specifically, the 2‐oxoethyl linker was extensively employed to connect the cinnamic acid core with remote TYR‐interacting groups. Interestingly, among several 2‐(cinnamoyloxy)acetic esters examined, compound **14** reached the nanomolar range (IC_50_=0.017 μM),^[55]^ while closely related analogues with methoxy and/or formyl substitutions at the terminal phenyl ring were three orders of magnitude less potent (IC_50_=24–43 μM).[^56^, ^57^] Two analogous 2‐(cinnamoyloxy)acetic amides, featuring *para*‐ and *meta*‐acetyl substituents, displayed TYR inhibition activity in the single‐digit nanomolar range (IC_50_=0.0020 μM for **15**),[^58^, ^59^] while compound **16**, which incorporates an additional methylene linker, was reported with a remarkable IC_50_ value of 0.000060 μM (0.06 nM) against mTYR, demonstrating a mixed‐type inhibition.^[60]^ In addition to these modulations, saturated analogues of simple cinnamic acid derivatives were evaluated. Compound **17**, the reduced direct analogue of **10**, was slightly more active than its parent (IC_50_=1.85 μM *vs*. 7.8 μM), while ester **18** provided an IC_50_ value (0.07 μM) consistent with previously mentioned esters and amides of cinnamic acid.^[61]^ Thus, the presence of the double bond did not appear to significantly influence the recorded activities. Finally, dimerization endeavours led to a series of compounds exhibiting two symmetrical resorcinol moieties, including compounds **19** and **20**, that were identified as competitive inhibitors of mTYR (IC_50_=0.65 μM and 0.78 μM, respectively).^[62]^

### 3.3 Stilbenoids and 1,2‐Diphenyl Compounds

**Figure 5:**
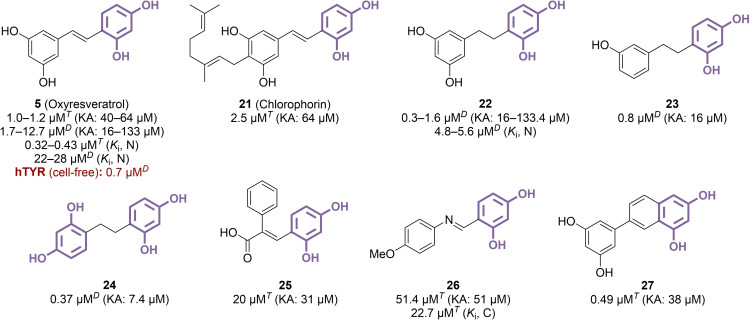
Stilbenoid derivatives as TYR inhibitors (C: competitive inhibition, N: non‐competitive inhibition, *T*: l‐tyrosine used as substrate, *D*: l‐DOPA used as substrate).

Replacing the carboxyl moiety by an additional phenyl ring shifted the molecular class from cinnamic acid to stilbenoid derivatives. Among the well‐examined resorcinol‐based TYR inhibitors utilizing a stilbene framework is oxyresveratrol **5**, a hydroxylated analogue of the renowned natural product resveratrol, featuring a 2,4‐dihydroxy‐type resorcinol along with a 3,5‐dihydroxy pattern that is not anticipated to interact with the TYR copper centre. This compound, frequently isolated from Moraceae species rich in resorcinol‐type polyphenols,^[63]^ has been repeatedly highlighted as a potent mTYR inhibitor in phytochemical studies, with IC_50_ values between 1.0 and 12.7 μM (Figure 5).[^64^, ^65^, ^66^, ^67^, ^68^] Notably, although it features a copper‐interacting resorcinol core, **5** was consistently classified as a non‐competitive inhibitor, indicating its probable binding to an allosteric site on mTYR.[^64^, ^65^] The compound also demonstrated an efficient hTYR inhibition activity (IC_50_=0.7 μM).^[68]^ Extracted from *Artocarpus xanthocarpus* with compound **5**, the geranyl analogue chlorophorin **21** exhibited comparable activity against mTYR (IC_50_=2.5 μM),^[66]^ suggesting a tolerance for additional substituents at the 3,5‐dihydroxyphenyl ring, and TYR binding through the other 2,4‐dihydroxyphenyl side. Saturated dihydrostilbene derivatives were especially investigated, as compound **22** afforded slightly better inhibition values than the direct analogue **5** (IC_50_=0.3–1.6 μM *vs*. 1.7–12.7 μM).[^65^, ^67^] Thus, modulations of **22** were undertaken, and among the tested compounds, only **23** showed a high level of activity.^[67]^ Derivatives with 4‐methoxy, 4‐fluoro or 2‐chloro substitutions, and particularly 1,2‐diphenylethanone counterparts, resulted in a substantial drop of TYR inhibitory efficacy (IC_50_ >30 μM).^[69]^ However, promising outcomes emerged when replacing the 3,5‐dihydroxyphenyl moiety with a 2,4‐dihydroxyphenyl group, which is better suited as a TYR‐targeting moiety. Indeed, symmetrical compound **24** was found as an efficient inhibitor of mTYR (IC_50_=0.37 μM). In general, glycosylated versions of the aforementioned inhibitors yielded mixed results in terms of TYR inhibition. While analogues of **24** incorporating xylose, glucose, cellobiose or maltose units mostly retained the initial potency (IC_50_=0.43–1.6 μM *vs*. 0.37 μM),[^70^, ^71^] the introduction of glucosyl moieties into compounds **5** or **22** resulted in a significant decrease in activity (IC_50_=29–231 μM, and 2.6–12.8 μM, respectively).[^72^, ^73^] Unsurprisingly, glycosylating the 2,4‐dihydroxyphenyl part was especially detrimental, underscoring its crucial importance for TYR binding. Combining the stilbene and cinnamic acid frameworks did not yield beneficial results (IC_50_=20 μM for **25**),^[74]^ nor did replacing one carbon atom of the central double bond with a nitrogen (IC_50_=51.4 μM for **26**).^[75]^ However, the addition of an extra six‐membered ring around the double bond proved to be successful, providing compound **27** with an IC_50_ value of 0.49 μM.^[76]^ This compound heralds the potential of flavonoid and isoflavonoid TYR inhibitors, that will be discussed further below.

### 3.4 Chalconoids and 1,3‐Diphenyl Compounds

**Figure 6:**
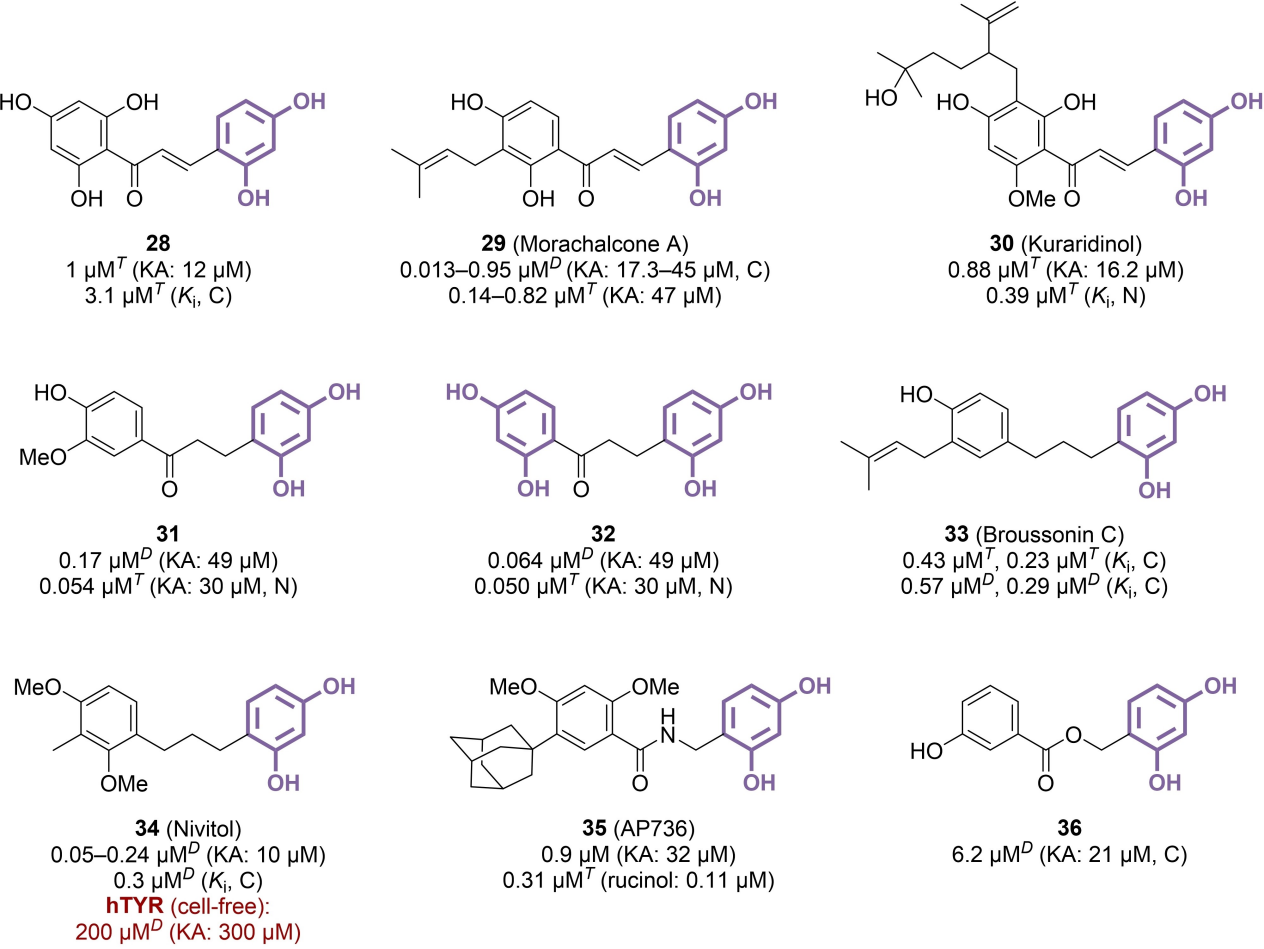
Chalconoid derivatives as TYR inhibitors (C: competitive inhibition, N: non‐competitive inhibition, *T*: l‐tyrosine used as substrate, *D*: l‐DOPA used as substrate).

Chalcones are naturally occurring products that can be viewed as higher homologues of the previously described stilbenoids, featuring an additional carbonyl‐type spacer between the two phenyl rings. Several chalcone derivative, both of natural and synthetic origins, were thus tested for their TYR inhibition potency. For example, compound **28** and its 2’‐dehydroxy analogue were identified among several other polyphenolic chalcone derivatives as resorcinol‐based TYR inhibitors, with IC_50_ values of 1 μM and 0.08–5 μM, respectively (Figure 6).[^77^, ^78^] Assessing the impact of an additional, fused phenyl ring to afford a naphthylchalcone was disappointing, leading to an IC_50_ of 14.4 μM.^[79]^ Similar to stilbenoids, many natural polyphenolic chalcones contain prenyl and/or geranyl components, with certain examples showing potent TYR inhibition. Notably, morachalcone A (**29**) and kuraridinol (**30**), isolated from *Morus*,[^80^, ^81^, ^82^] *Artocarpus*[^83^, ^84^] and/or *Sophora* ^[85]^ genera, demonstrated nanomolar IC_50_ values ranging from 0.013 to 0.95 μM, albeit with differing inhibition mechanisms. Specifically, compound **29** was identified as a competitive inhibitor, whereas compound **30** acted as a non‐competitive inhibitor. The reduction of the double bond also afforded valuable compounds through synthetic ways. Dihydrochalcones **31** and **32** illustrate the potential of these more flexible derivatives, achieving IC_50_ values below 100 nM.^[86]^ Compound **32** especially showed effective skin permeation and melanogenesis suppression properties in a guinea pig model, indicating its ability to act *in vivo* and to undergo further dermocosmetic development. The additional reduction of the carbonyl group to yield resorcinol‐based compounds with a saturated 1,3‐diphenylpropane scaffold did not abolish the TYR inhibition activity as well. In particular, *Broussonetia kazinoki* contained several prenylated analogues of 1,3‐diphenylpropane, among which competitive inhibitor **33** (broussonin C, IC_50_=0.43–0.57 μM) stood out as the most promising.[^87^, ^88^] Originally extracted from *Dianella ensifolia*, nivitol (**34**) is another example of 1,3‐diphenyl compound with a propane‐type linker. While its activity against mTYR aligned with previously mentioned analogues (IC_50_=0.05–0.24 μM), **34** demonstrated much lower inhibitory potency against the human enzyme (IC_50_=200 μM).[^28^, ^89^, ^90^] Nevertheless, a 0.1 % formulation of this compound was capable of inhibiting melanogenesis in reconstructed human skin with similar efficacy to 1 % kojic acid.^[89]^ This could be attributed to the ability of **34** to accelerate hTYR degradation, alongside its modest inhibition of the enzyme's catalytic function.^[90]^ Furthermore, attempts were made to explore the potential of amide and ester counterparts of resorcinol dihydrochalcones. Regarding amides, while initial results from a study focusing solely on polyhydroxylated compounds were discouraging,^[91]^ the addition of methyl, methoxy, and particularly adamantyl groups proved to be highly advantageous. Among several submicromolar inhibitors, compound **35** (AP736) emerged as the most promising, in terms of mTYR inhibition (IC_50_=0.9 μM) and B16 murine melanoma cells melanogenesis decrease (IC_50_=1.1 μM).^[92]^ Clinical studies were conducted using a 0.5 % formulation of **35**, resulting in a significant reduction of facial hyperpigmentation after 3 and 6 weeks.^[93]^ Subsequent research proposing the replacement of the adamantyl group with various alternatives revealed the broad substituent tolerance at this position, as all derivatives were potent inhibitors (IC_50_=0.05–0.19 μM).^[94]^ Switching to esters only afforded moderately active benzyl benzoate compounds (IC_50_=6.2 μM for **36**), as only polyhydroxylated derivatives were tested, once again underscoring the importance of inserting a hydrophobic component in inhibitor design.^[95]^

### 3.5 Flavonoids and Isoflavonoids

**Figure 7:**
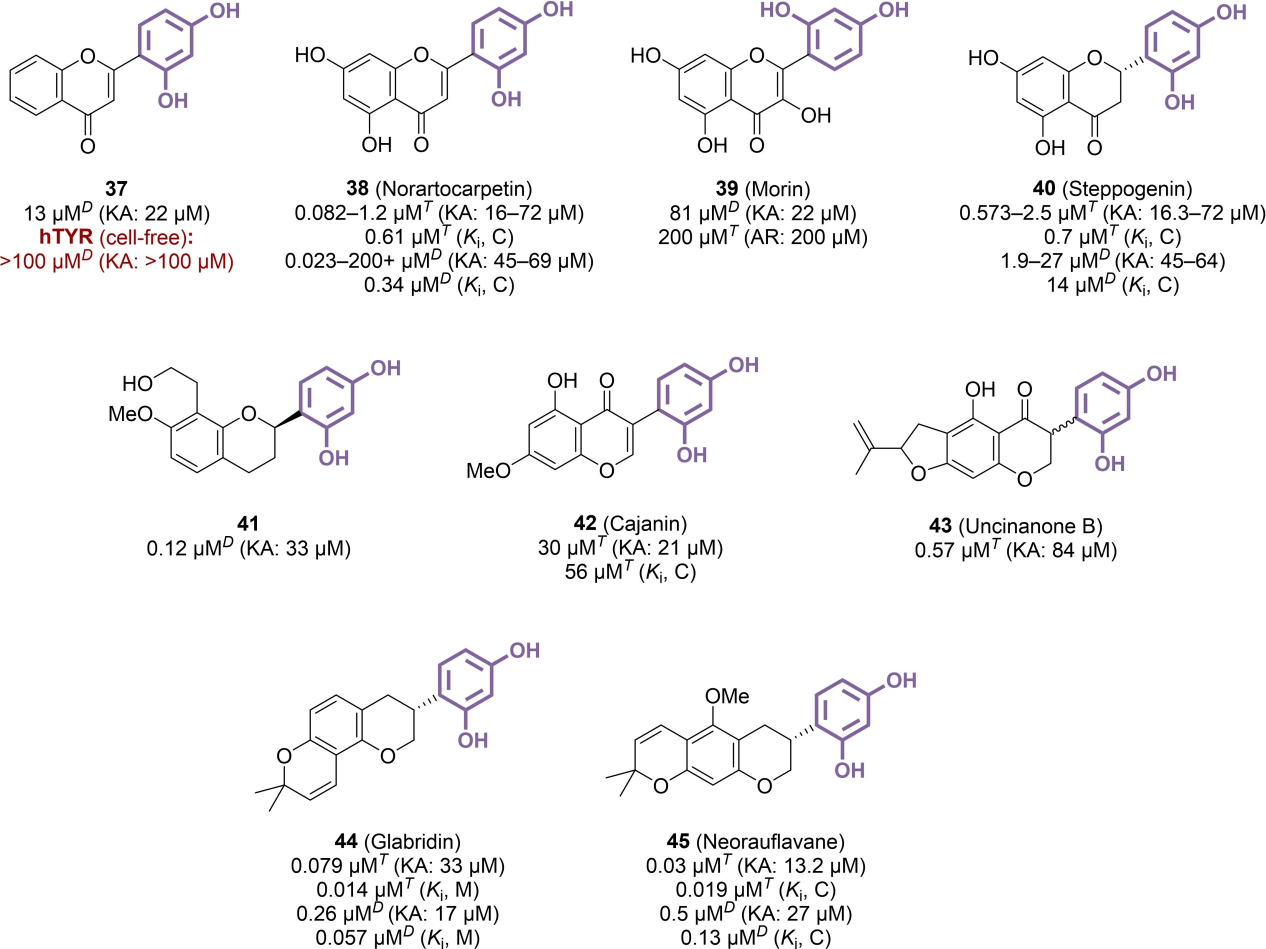
Flavonoid and isoflavonoid derivatives as TYR inhibitors (C: competitive inhibition, M: mixed‐type inhibition, *T*: l‐tyrosine used as substrate, *D*: l‐DOPA used as substrate).

Plethoric phytochemical studies have identified resorcinol‐based flavonoids from specific Moraceae species, especially belonging to *Morus* and *Artocarpus* genera, as TYR inhibitors. However, their inhibitory activities were generally weak, with structural differences mainly due to the type and placement of glycosyl and prenyl‐like groups, and with frequent reevaluations of already reported products. Consequently, the focus of this discussion will be on the structural modifications to the flavone core and how these alterations influence tyrosinase inhibition efficacy, rather than providing a comprehensive inventory of tested analogues. First, it is worth noting that the simplest resorcinol‐containing flavone, compound **37**, did not appear as a very potent mTYR inhibitor, and had no significant effect on the human enzyme in MNT‐1 lysates (Figure 7).[^31^, ^96^] However, naturally occurring analogues with a 5,7‐dihydroxy substitution pattern, such as norartocarpetin **38**, showed more promising results, although there were considerable variations across different assays. Indeed, its IC_50_ ranged from 0.082 μM to 1.2 μM when using l‐tyrosine as a substrate,[^97^, ^98^, ^99^, ^100^] while switching to l‐DOPA provided values from 0.023 μM to over 200 μM,[^101^, ^102^, ^103^] a >10,000‐fold difference that compromises the evaluation of its true efficacy. If the introduction of a hydroxy group at position 3 to afford the corresponding flavonol **39** (morin) resulted in a significant drop in activity,[^105^, ^106^] reducing the endocyclic double bond provided flavanone **40** (steppogenin) which exhibited a more favourable inhibitor profile.[^66^, ^97^, ^98^, ^101^, ^106^, ^107^] Compounds with reduced carbonyl, corresponding to the flavan subfamily, were explored especially through isolation from natural sources. Among three compounds active against mTYR at the submicromolar range, compound **41** displayed the best IC_50_ value (IC_50_=0.12 μM).^[108]^ Consequently, the core structure of flavones did not seem to inherently enhance TYR inhibition: in fact, progressively removing its defining features generally maintained or improved inhibitory performance. This trend was similarly observed with modifications to the isoflavone core structure. Indeed, while natural product **42** (cajanin) was moderately active against mTYR (IC_50_=30 μM),^[109]^ reduced analogues, such as **43** (uncinanone B, IC_50_=0.57 μM) or desmodianone H, allowed a consequent improvement.^[110]^ Like with flavones, completely reduced derivatives showed an overall activity increase. The potency of **44** (glabridin), a compound isolated from *Glycyrrhiza glabra*, is an emblematic illustration, as the compound was extensively studied for its TYR inhibition properties, and is currently used as a skin‐whitening ingredient in dermocosmetic preparations. Although it was generally found as an efficient mTYR inhibitor (IC_50_=0.079–18.6 μM),[^111^, ^112^, ^113^] data on its impact on hTYR and human skin remain scarce. Another resorcinol‐containing flavan, compound **45** (neorauflavane) yielded interesting results with IC_50_ values in a similar range (IC_50_=0.03–0.5 μM).^[114]^ However, despite the potency of these specific compounds, the majority of resorcinol flavonoids did not emerge as particularly appealing inhibitors, and their predominance in TYR inhibition‐related literature likely stems from the myriad phytochemistry studies describing them, rather than a genuine scaffold superiority.

### 3.6 Hemiindigoids

**Figure 8:**
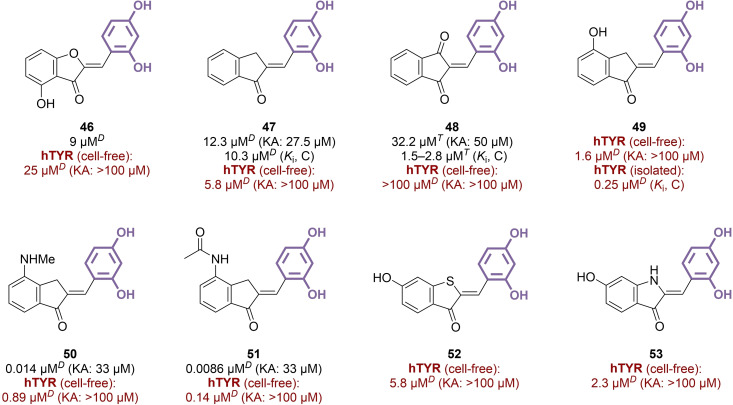
Hemiindigoid derivatives as TYR inhibitors (C: competitive inhibition, *T*: l‐tyrosine used as substrate, *D*: l‐DOPA used as substrate).

Hemiindigoids are compounds that could be seen as isomers of flavones, with a central five‐membered ring and an exocyclic double bond. The intracyclic atom at position 3 can vary, classify them into aurones (O), indanones (CH_2_), hemiindigos (NH) and hemithioindigos (S).^[115]^ Although the resorcinol‐based hemiindigoid scaffolds did not provide more promising results than flavones and isoflavones against mTYR, they garnered significant attention for their anti‐hTYR activities. Compound **46**, among other aurones, underwent comparative testing against mTYR, hTYR (from MNT‐1 cell lysates) and TYRs from *Streptomyces antibioticus* and *Polyporus arcularius*, yielding modest results in the same order of magnitude (IC_50_=4–34 μM, Figure 8).[^31^, ^116^, ^117^] Transitioning to the naked resorcinol‐indanone scaffold **47** and its 3‐oxo analogue **48** did not substantially improve both mTYR and hTYR inhibition activities (IC_50_=5.8–100+μM).[^31^, ^118^, ^119^] However, substituents at positions 4 and 5 demonstrated considerable benefits in some cases, as testified by the potent competitive inhibitory activity of 4‐hydroxy compound **49** on hTYR, both in its pure form (*K*_i_=0.25 μM) and in cell lysates. Indanone **49** also suppressed melanogenesis in human MNT‐1 melanoma whole cells, with an IC_50_ value of 29 μM. Its aurone counterpart displayed similar potential, indicating minimal influence from the atom present at position 2.^[31]^ Further modulations at position 4 were undertaken, resulting in the synthesis of a series of resorcinol‐containing 4‐amino and 4‐amidoindanones. Compounds **50** and **51** were found as the most promising derivatives.^[120]^ While **51** appeared as the most efficient inhibitor of both mTYR and hTYR (IC_50_=0.0086 μM and 0.14 μM, respectively), **50** was more active in a cellular context, leading to an IC_50_ for melanogenesis suppression as low as 0.77 μM after 14‐day treatment of MNT‐1 cells, a value comparable to that of reference thiamidol (IC_50_=0.28 μM), and significantly better than kojic acid (IC_50_=800 μM). Atom substitution at position 3 yielded for example sulfur‐ and nitrogen‐based compounds **52** and **53** with a comparable profile than analogous aurones and indanones. However, further alterations of the hemiindigoid scaffold, such as carbonyl reduction or nitrogen insertion at position 2, inevitably resulted in a dramatic loss of activity against hTYR.^[31]^

### 3.7 Benzylidene Hydrazides and Azines

Benzylidene hydrazides represent another recurring category of resorcinol derivatives aimed at inhibiting tyrosinase, consisting of a C=N bond connecting an aromatic part, which includes the resorcinol group, to variously substituted hydrazide units. The initial examples of this class, specifically compounds **54** and **55**, featured a thiosemicarbazide group known for its interaction with the dicopper centre of TYR, making them heterodivalent inhibitors (Figure 99).[^121^, ^122^] Consequently, their inhibitory effectiveness (IC_50_=0.18 μM for **54** and 0.58 μM for **55**) raised questions about whether the resorcinol or thiosemicarbazide components were the primary contributors to their potency. The production of a *N*‐methyl derivative of **55**, compound **56**, suggested a significant role of the thiosemicarbazide portion, as evidenced by the substantial decrease in inhibitory capability upon methylation (IC_50_=6 μM for **56**, a tenfold reduction).^[123]^ Attempts to develop hydrazide derivatives through extended substitution patterns led to a series of compounds whose effectiveness seemed not to stem mainly from the resorcinol component. Instead, variations in the phenyl ring, such as halogen substitutions, proved more advantageous for activity, indicating a probable interaction with TYR mediated by different elements of the molecular structure. For instance, compound **57** and its demethylated variant were identified as relatively ineffective against mTYR, with IC_50_ values of 32 μM and 37 μM, respectively.[^124^, ^125^] Explorations into heterocyclic substituents, including imidazopyridine and indole‐based derivatives, resulted in a modest uptick in inhibitory efficacy (IC_50_=13 μM and 3.3 μM for compounds **58** and **59**, respectively).[^126^, ^127^] Nonetheless, these figures remained in the micromolar range and did not approach the levels of the most potent resorcinol‐derived TYR inhibitors documented. In the case of polyphenolic compound **60**, the resorcinol segment seemed to exert a more pronounced influence on its activity, though it did not enhance global effectiveness (IC_50_=3.3 μM).^[128]^ However, compound **60** demonstrated the ability to reduce browning in fresh‐cut apples, and prevent shrimp deterioration, as evidenced by favourable pH and bacterial counts after 6 days. Replacing the hydrazide moiety with a symmetrical benzylidene azine did not yield significant improvements either, and compound **61** was associated with an IC_50_ value of 7 μM.^[129]^

**Figure 9:**
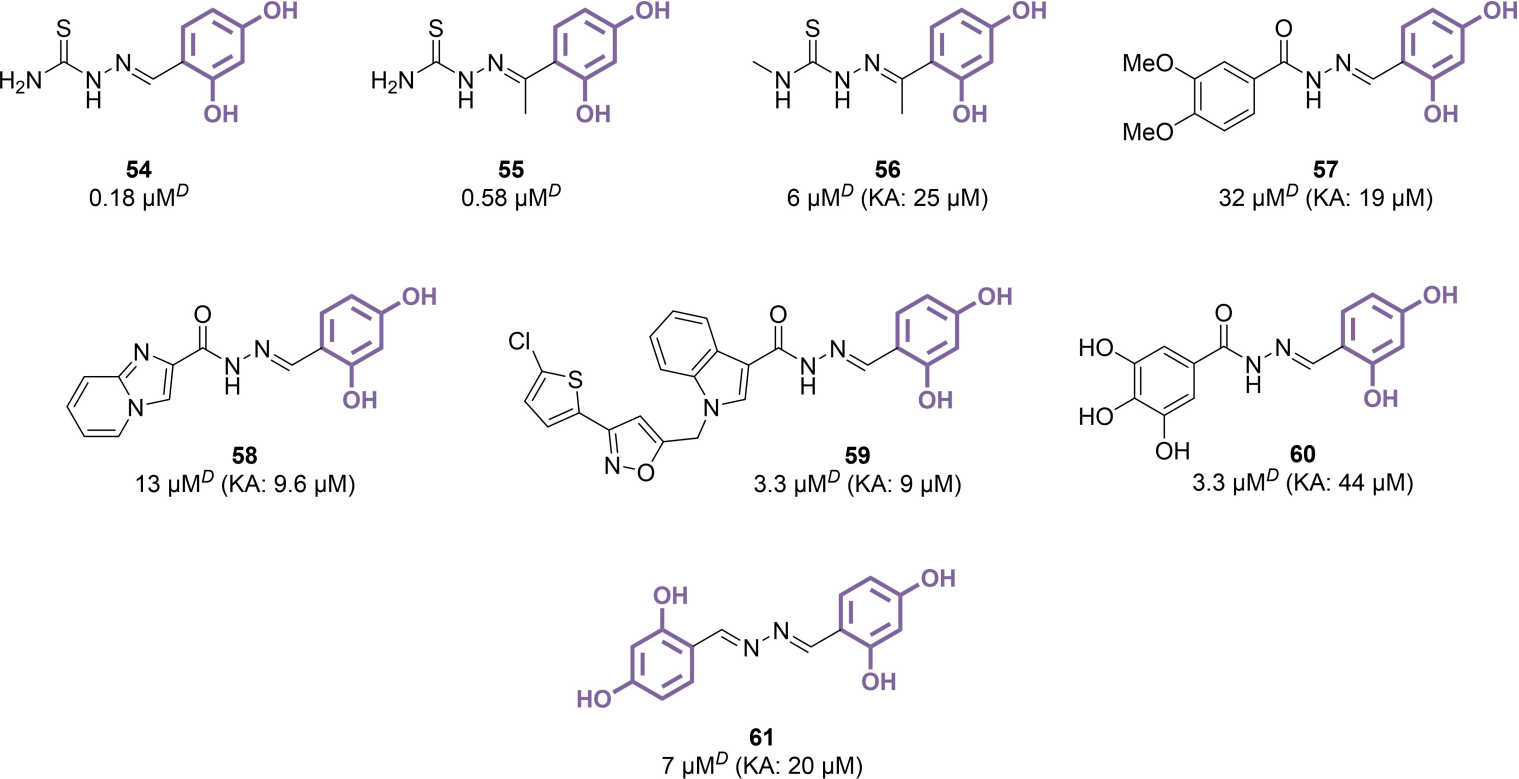
Benzylidene hydrazide and azine derivatives as TYR inhibitors (*D*: l‐DOPA used as substrate).

### 3.8 Benzylidene Heterocyclic Derivatives

**Figure 10:**
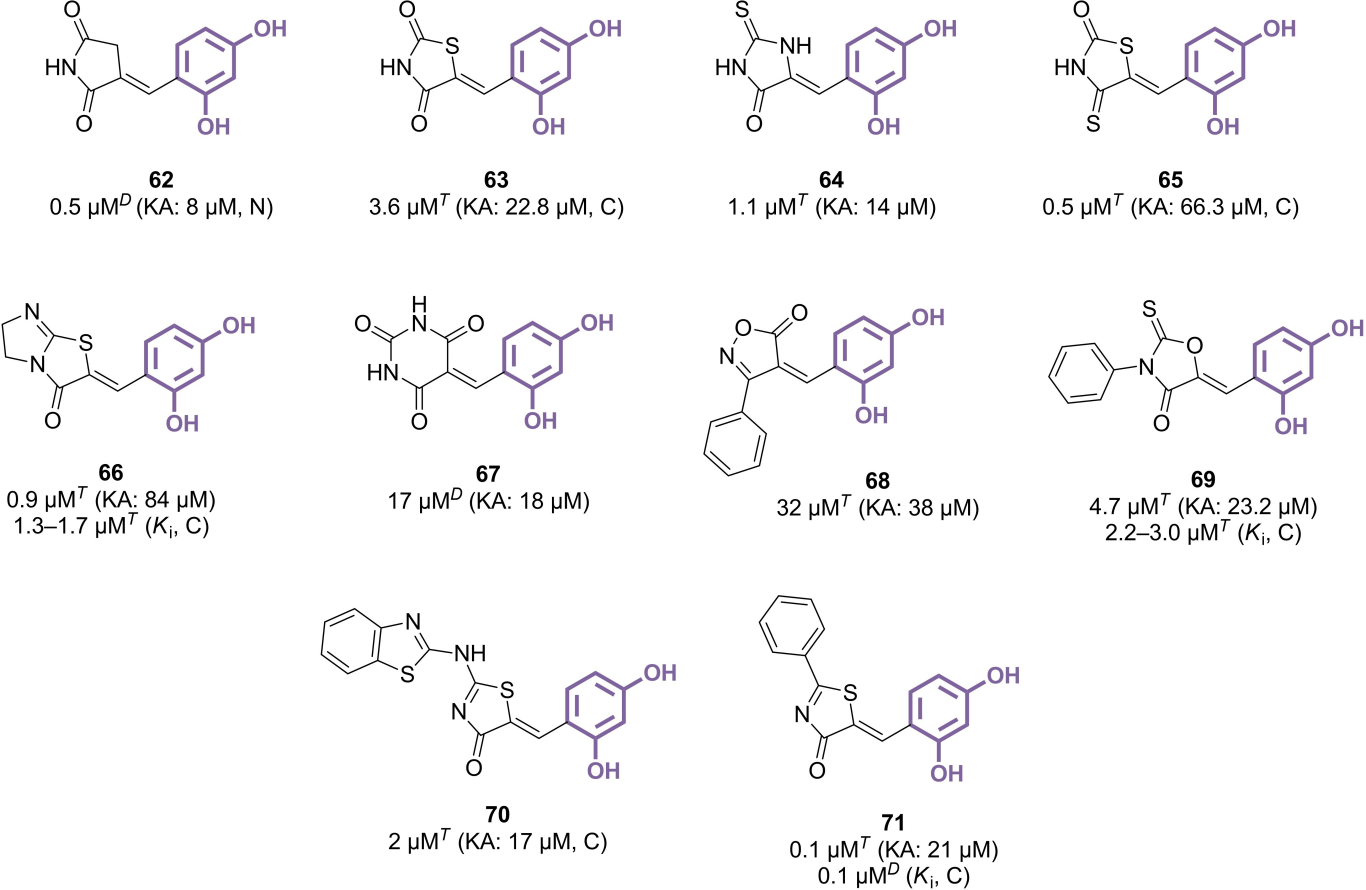
Benzylidene heterocyclic derivatives as TYR inhibitors (C: competitive inhibition, N: non‐competitive inhibition, *T*: l‐tyrosine used as substrate, *D*: l‐DOPA used as substrate).

Using the benzylidene link to attach the resorcinol‐containing phenyl part to various five‐membered heterocyclic rings could be viewed as an expansion of the hemiindigoid strategy, which globally appeared as more promising than the previously described hydrazide‐based approach. Pyrrolidine‐2,5‐dione compound **62**, the first reported analogue, achieved submicromolar activity (IC_50_=0.5 μM), with a surprising non‐competitive inhibition mechanism (Figure 10).^[130]^ Encouraging results were also observed *in vivo* using HRM2 hairless mice, where treatment with at least 2 μM of **62** reduced UVB‐induced pigmentation. Subsequent investigations explored the replacement of various atoms within the heterocyclic part. The introduction of an intracyclic sulfur to form the thiazolidine‐2,4‐dione counterpart **63** proved detrimental (IC_50_=3.6 μM), although it resulted in a competitive inhibitor.^[131]^ Switching the carbonyl groups into thiocarbonyls had only minimal impact (IC_50_=0.5–1.1 μM), whether using hydantoin (**64**) or thiazolidine‐2,4‐dione (**65**) scaffolds.[^132^, ^133^] By expanding the heterocyclic framework, the bicyclic compound **66** was identified, which bears resemblance to the previously mentioned hemithioindigos. This compound was active in the submicromolar range (IC_50_=0.9 μM) and demonstrated a moderate inhibitory effect on melanogenesis in murine melanoma B16F10 cells.^[134]^ For each of these examples, chemical modulation was performed at the phenyl ring, and the 2,4‐dihydroxy analogue consistently showed superior efficacy, thereby indicating a crucial role of the resorcinol moiety. This finding starkly contrasts with the observations made for hydrazide derivatives. However, the relatively modest activity achieved by barbiturate counterpart **67** suggested that using a six‐membered ring might not be advantageous at this position.^[135]^ Consequently, further endeavours focused on substituting five‐membered rings at different positions. While introducing a phenyl substituent on an isoxazolone scaffold, positioned in α of the benzylidene part, resulted in a loss of inhibitory activity against mTYR (IC_50_=32 μM for compound **68**),^[136]^ relocating this group as a *N*‐substituent in an oxazolinone ring produced the more potent compound **69**, with an IC_50_ of 4.7 μM.^[137]^ The position 2 of thiazolone heterocycles emerged as the most promising for substitution, foreshadowing studies around a directly linked thiazole core that will be developed in the next section. After a first work leading to the discovery of compound **70** as a mTYR inhibitor with an IC_50_ value of 2 μM,^[138]^ three recent studies described analogous compounds featuring either a 2‐benzylamino or a 2‐phenyl group, resulting in improved competitive inhibition properties (IC_50_=0.27 μM and 0.1 μM for the 2‐benzylamino derivative and compound **71**, respectively).[^139^, ^140^, ^141^] Intriguingly, both molecules were also found to act as inhibitors of the expression of TYR and/or melanogenesis‐associated proteins, in addition to their direct action on the enzyme's catalytic activity.

### 3.9 Thiamidol and Directly Linked Heterocycles

**Figure 11:**
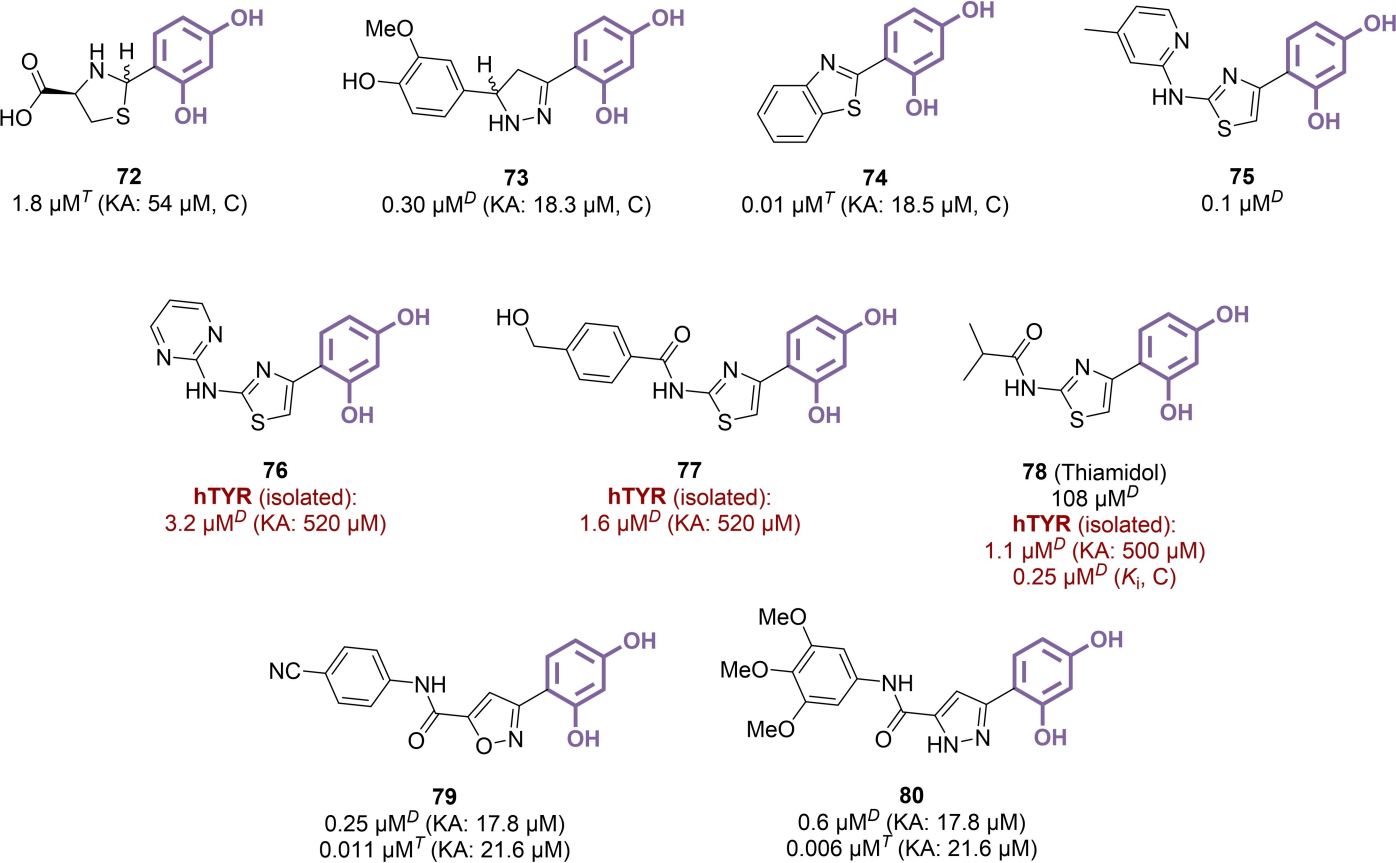
Heterocycles directly linked to a resorcinol moiety as TYR inhibitors (C: competitive inhibition, *T*: l‐tyrosine used as substrate, *D*: l‐DOPA used as substrate).

**Figure 12:**
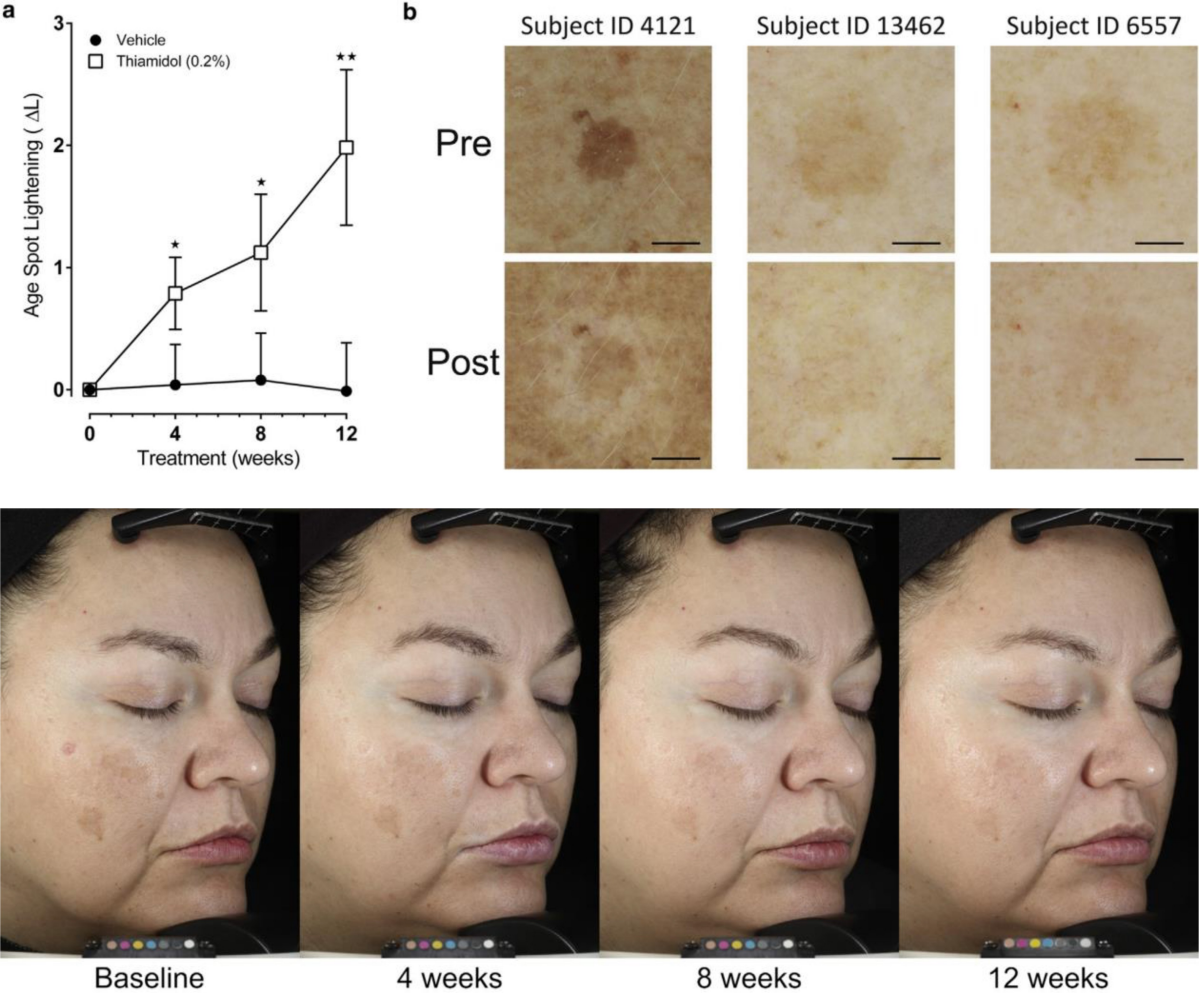
Effect of a 0.2 % thiamidol preparation on age spots in preliminary clinical studies (upper panel). Images of a patient with facial melasma at baseline and after 4, 8 and 12 weeks of treatment with a 0.2 % thiamidol prepration (lower panel). Reproduced from ref [28, 148] (Creative Commons, CC BY‐NC‐ND 4.0 license).

Removing the benzylidene linker provided resorcinol moieties directly linked to heterocyclic scaffolds, mainly azoles.^[142]^ Compound **72**, featuring a saturated thiazolidine ring, remained only moderately active (IC_50_=1.8 μM),^[143]^ while unsaturated and aromatic analogues displayed greater potential as mTYR inhibitors (Figure 11). Indeed, phenyl‐substituted dihydropyrazole **73**, and especially fully aromatic benzothiazole **74**, were found as more promising compounds, with submicromolar activity (IC_50_=0.30 μM and 0.01 μM, respectively), highlighting the advantage of directly linking these moieties with the resorcinol part.[^144^, ^145^] All three derivatives were associated with a competitive inhibition mechanism as expected. However, the most extensively studied heterocyclic series revolved around a 2‐aminothiazole core, following the early identification of compound **75**, which showed good potency against mTYR (IC_50_=0.1 μM) and effectively reduced pigmentation in MNT‐1 human melanoma cells (83 % at 15 μM).^[146]^ A more recent study relied on this heterocyclic framework to inhibit hTYR isolated from TYR‐expressing HEK‐293 cells. Among the tested compounds, **76** and **77** (IC_50_=3.2 μM and 1.6 μM against hTYR) represent the two main subfamilies studied, *i. e*. 2‐amines and 2‐amides, with generally better outcomes obtained in the latter category.^[147]^ The most active compound was isobutylamido derivative **78** (thiamidol), whose efficacy was further explored through *K*_i_ determination (*K*_i_=0.25 μM).^[28]^ In addition, thiamidol produced good results in a MelanoDerm skin model (IC_50_=0.9 μM) and in an initial clinical investigation, revealing a significant skin‐lightening effect on age spots following a 4‐to‐12‐week treatment with 0.2 % of the compound (Figure 12). These positive findings were corroborated by several studies focusing on clinical assessment of thiamidol in the treatment of facial melasma or post‐inflammatory hyperpigmentation. In one study involving women with mild‐to‐moderate melasma, a split‐face randomized trial was conducted, administering 0.2 % thiamidol treatment on one side of the face and 2 % hydroquinone treatment on the other.^[148]^ The thiamidol‐treated side exhibited a significantly improved modified Melasma Area and Severity Index (mMASI) compared to the hydroquinone‐treated side (−36 % *vs*. −19 % after 12 weeks), while the latter resulted in a worsening of this score in 10 % of cases. In another study comparing 0.2 % thiamidol and 4 % hydroquinone preparations for facial melasma treatment, similar outcomes were observed (mMASI change of −43 % *vs*. −33 % after 12 weeks).^[149]^ However, this time, mild adverse effects were reported for thiamidol, and two cases of allergic contact dermatitis were noted among fifty patients. When applied to moderate‐to‐severe forms of melasma for 24 weeks, thiamidol also yielded favourable results, resulting in a significantly improved mMASI reduction (−26 %) compared to the vehicle (−14 %).^[150]^ However, at 13–20 weeks after treatment cessation, the initial index was partially restored, probably due to reversible hTYR inhibition. Thiamidol demonstrated comparable performances as a treatment for post‐inflammatory hyperpigmentation in another dedicated clinical trial.^[151]^ The compound inspired further work revolving around resorcinol moieties directly linked to amido‐substituted heterocycles. A recent study described series of TYR‐targeting molecules based on oxazole and diazole scaffolds, yielding for examples compounds **79** and **80**, which are low nanomolar mTYR inhibitors (IC_50_=0.006–0.011 μM when using l‐tyrosine as substrate).^[152]^ These compounds were evaluated as potential neuromelanogenesis suppressors, with possible applications in the treatment of Parkinson's disease (PD). In this context, **80** was able to alleviate movement dysfunctions in mice with methyl‐4‐phenyl‐1,2,3,6‐tetrahydropyridine‐induced PD symptoms, with a similar efficacy than reference drug benserazide.

## 4 Interaction Patterns with hTYR and mTYR

**Figure 13:**
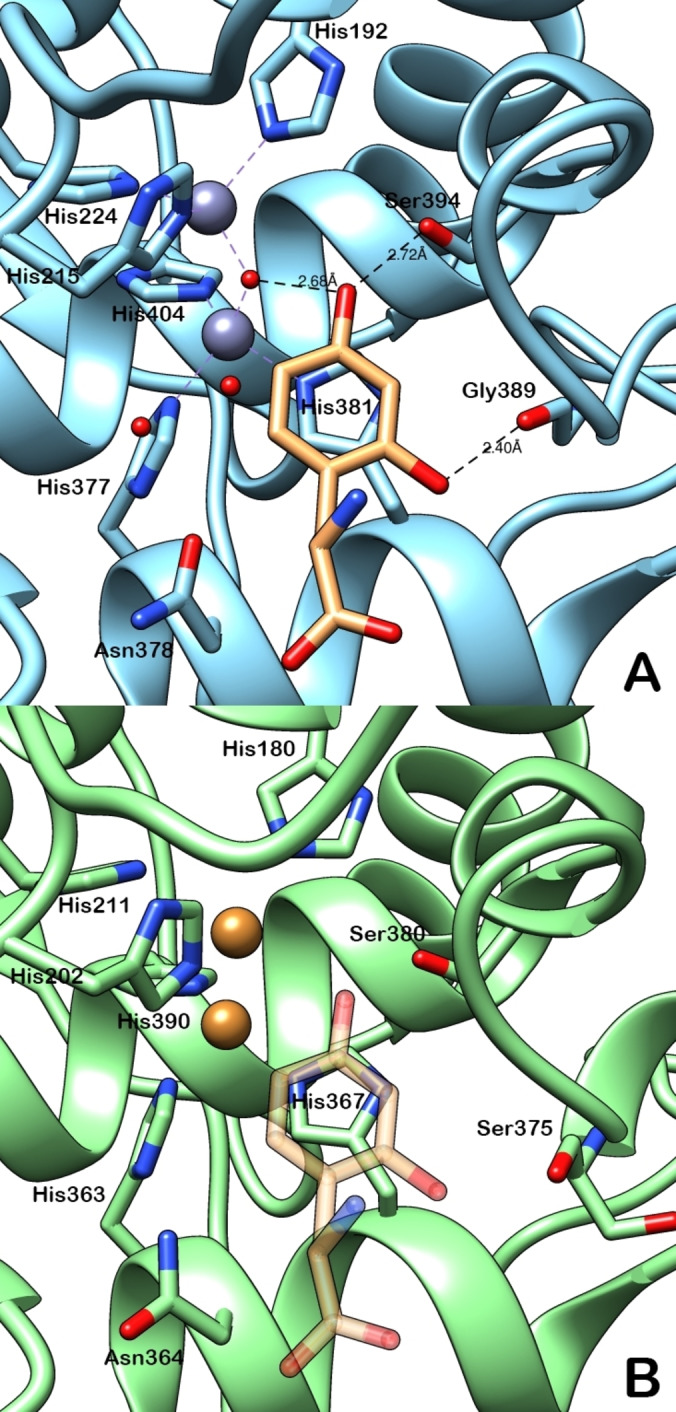
(A) Crystal structure of a complex including TRP‐1 and 2,4‐dihydroxyphenylalanine (PDB 9EY7). (B) Superimposition of the previous structure with a homology model of hTYR (only the ligand was retained from 9EY7).

Numerous efforts have been made to unravel the key structural features of resorcinol‐TYR interactions, particularly considering the reported differences between the human and mushroom versions of the enzyme. These investigations were challenged by the lack of a proper hTYR crystal structure and the general difficulties in co‐crystallizing ligands with TYR enzymes. However, recent computational studies have provided valuable insights. Notably, in 2023, two independent studies using docking and molecular dynamics with thiamidol (compound **78**) yielded remarkably similar results regarding the role of the resorcinol ring in binding to hTYR.[^153^, ^154^] In these simulations, the *para*‐hydroxy group of the resorcinol was observed to coordinate with copper atom Cu_A_ and form a hydrogen bond with Ser380, similar to interactions reported for compounds **49** and **51** for example.[^31^, ^120^] Additionally, the *ortho*‐hydroxy group of the resorcinol ring was involved in a hydrogen bond with the carbonyl oxygen of Asn364. Interestingly, other computational studies on resorcinol‐containing compounds like **5** and **49** indicated the formation of a hydrogen bond with Ser375 instead.[^31^, ^68^] In fact, Asn364 and Ser375 are residues located on either side of the second coordination sphere and may engage with the resorcinol ring depending on the orientation of the *ortho*‐hydroxy group (Figure 13B). Furthermore, the resorcinol fragment backbone was found to interact with Val377 and His367, the latter through π‐π stacking, as observed for most of the phenolic scaffolds binding to the dicopper centre. Recently, a crystal structure of a resorcinol‐based modified phenylalanine complexed with TRP‐1, an enzyme highly homologous to hTYR but containing zinc ions instead of copper, provided experimental confirmation of the theoretical predictions (Figure 13 A).^[155]^ The resorcinol moiety exhibited a similar interaction pattern: the *para*‐hydroxy group coordinated with the metal ions and Ser394 (analogous to Ser380 in hTYR), while the *ortho*‐hydroxy group formed a hydrogen bond with Gly389 (equivalent to Ser375 in hTYR). Additionally, His381 stabilized the resorcinol ring through π‐π stacking, akin to His367 in hTYR‐based calculations. These findings support the validity of theoretical studies conducted with hTYR. In contrast, computational data reported for mTYR are considerably more inconsistent, and the lack of a crystal structure of a resorcinol‐mTYR complex further complicates interpretation. A key difference between mTYR and hTYR is the absence of a serine residue to anchor the *para*‐hydroxy group of resorcinols, as the corresponding residues in the PPO3 and PPO4 isoforms of mTYR are alanines. This has led to varying reports on the interaction patterns of thiamidol with the enzyme.[^153^, ^154^] Predictions include hydrogen bonding between the *ortho*‐hydroxy group and either Met280 or Asn260, with broader contacts reported between the resorcinol fragment and residues such as Val283 and Ala286, or Val283, His259, and His263. Moreover, numerous helter‐skelter docking studies using mTYR crystal structures and resorcinols exist in the literature, producing contradictory results that contribute more to confusion than to a clear understanding of the favoured interaction mode. Clearly, further research is necessary to fully elucidate the structural determinants of mTYR inhibition by resorcinols.

## 5 Summary and Outlook

More than ever, resorcinol emerges as a privileged component for interacting with the TYR dicopper centre, thereby inhibiting the enzyme's catalytic activity. Initially, research primarily focused on simple derivatives centred around the resorcinol ring with appended side chains. However, efforts later transitioned to more intricate structures derived from natural products like flavonoids, cinnamic acids, or stilbenoids, and eventually to entirely synthetic scaffolds, such as benzylidene derivatives and directly liked heterocycles. Notably, the discovery of thiamidol marked a significant breakthrough in the search for effective inhibitors of human tyrosinase (hTYR). Thiamidol demonstrated remarkable activity against the isolated enzyme, and this efficacy was maintained in cellular and clinical settings, including in moderate‐to‐severe melasma cases. Resorcinol‐based compounds now constitute safer options among widely used skin‐whitening agents, particularly with compounds like thiamidol, rucinol, and alkylresorcinols, which are relatively innocuous. In contrast, alternative compounds such as kojic acid, hydroquinone, or arbutin are often associated with irritant, carcinogenic and/or genotoxic properties. However, the exact molecular mechanism by which resorcinols act on TYRs remains contentious. While some studies suggest that these compounds may function as alternative substrates or suicide inactivators through different mechanisms, other data support a role of true inhibitor, capable of safely operating within cellular environments without producing cytotoxic quinones. Further insights are needed to definitively address this topic by thoroughly examining the behaviour of various resorcinols when interacting with tyrosinases. Crystallographic data involving more complex and bioactive resorcinol‐containing compounds could provide valuable insights, potentially linking the interaction patterns with specific active site residues to the likelihood of these compounds to undergo TYR‐mediated oxidation. A similar approach was recently applied to other *ortho*‐substituted monophenols, using molecular docking studies.^[156]^ Future research should focus on both elucidating the mechanism of action of the resorcinol ring itself and developing more active and selective analogues based on alternative synthetic scaffolds. These next generations of compounds could serve as promising candidates for exploring uncharted territories where TYR activity could be implicated, like human brain. A first incursion was attempted recently in this direction with compounds **79** and **80**, opening promising horizons.

## Conflict of Interests

The authors declare no conflict of interest.

## Biographical Information

*Dr. Morane Beaumet obtained her PhD in Chemistry from Paris‐Saclay University (France) in 2022, under the supervision of Prof. Jean‐Pierre Mahy and Dr. Wadih Ghattas at the Laboratoire de Chimie Bioorganique et Bioinorganique of the Institut de Chimie Moléculaire et des Matériaux d'Orsay. There, she worked on the synthesis of artificial metalloenzymes (β‐lactamase and quercetinase). In the same year, she joined the Département de Pharmacochimie Moléculaire of University Grenoble Alpes as a postdoctoral associate, where she is developing tyrosinase inhibitors and hemiindigoid photoswitches*.

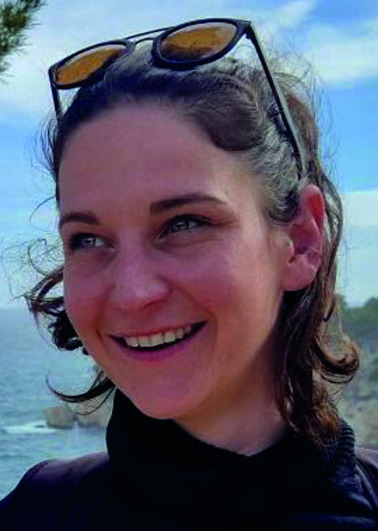

## Biographical Information

*Letícia M. Lazinski received her Bachelor of Pharmacy from the Federal University of Parana (Brazil) in 2016. She did her Master in Chemistry for Life Sciences at University Grenoble Alpes (France), where she started her Ph.D. in 2021 under the supervision of Dr. Romain Haudecoeur and Prof. Guy Royal. Her research work is axed on medicinal chemistry and photopharmacology, focusing on the development of tyrosinase inhibitors and photoswitchable acetylcholinesterase inhibitors*.

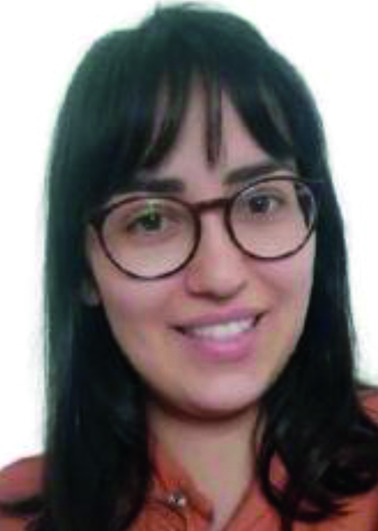

## Biographical Information

*Dr. Marc Maresca is researcher at the Aix‐Marseille Université. He received his PhD in Biochemistry at the Université Paul Cézanne (France, 2003) working on food contaminants named mycotoxins. He is currently working at the Institut des Sciences Moléculaires de Marseille (ISM2) UMR‐AMU‐CNRS 7313 and his research aims to identify and develop new molecules ‐ natural, synthetic, or bio‐inspired ‐ with therapeutic values, including antimicrobial peptides and their mimics as well as plant molecules and their derivatives*.

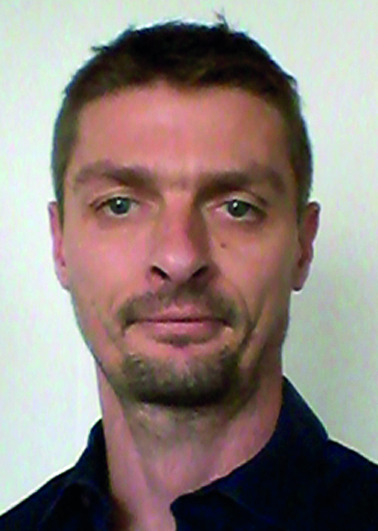

## Biographical Information

*Dr. Romain Haudecoeur obtained his PhD in Chemical Biology from University Grenoble Alpes (France) and University of Geneva (Switzerland) in 2011, under the supervision of Prof. Ahcène Boumendjel and Prof. Pierre‐Alain Carrupt. Then, he joined the group of Dr. David Monchaud at University of Burgundy (Dijon, France) as a postdoctoral associate. He was appointed in 2012 as Researcher in the Département de Pharmacochimie Moléculaire at University Grenoble Alpes and received in 2020 the Accreditation to Supervise Research (HDR) in Pharmacy. His research work primarily revolves around the rational targeting of biomacromolecules and biological functions with pharmacological and photopharmacological small‐molecule agents*.